# Establishment and application of high‐pressure propagation breeding (HPPB)‐mediated genetic transformation system in citrus rootstocks

**DOI:** 10.1111/pbi.70072

**Published:** 2025-04-29

**Authors:** Si‐Yu Zhang, Rui‐Fang Luo, Ya‐Xiao Wu, Ting‐Ting Zhang, Abdulhamid Yusuf, Nian Wang, Min Li, Shuo Duan

**Affiliations:** ^1^ Jiangxi Provincial Key Laboratory of Pest and Disease Control of Featured Horticultural Plants Gannan Normal University Ganzhou Jiangxi China; ^2^ Department of Plant Science and Biotechnology Federal University Dutsin‐Ma Katsina State Nigeria; ^3^ Citrus Research and Education Center, Department of Microbiology and Cell Science IFAS, University of Florida Lake Alfred FL USA

**Keywords:** citrus rootstocks, genetic transformation, *Agrobacterium rhizogenes*, high‐pressure propagation breeding (HPPB), rootstock improvement

Citrus cultivation plays a pivotal role in global agriculture and food security. With intensifying international market competition and increasing environmental challenges, citrus crops have become particularly urgent (Mukhametzyanov *et al*., 2024). Traditional breeding and genetic transformation are two main strategies for improvement, with the latter gaining more attention due to its ability to introduce specific traits that are difficult to achieve through conventional methods (Gutierrez‐E *et al*., 1997). Citrus rootstocks are crucial for enhancing fruit quality, disease resistance, and stress tolerance; their root systems are not only vital for water and nutrient uptake but also help establish beneficial connections with soil bacteria (Song *et al*., 2023). Genetic transformation technology offers tremendous potential for improving citrus crops without altering the genetic background of the scion (Cheng *et al*., 2021). This technology enhances rootstock quality, disease resistance, reduces pesticide use, improves fruit safety, and boosts market competitiveness (D'Amico *et al*., 2018; Zhang *et al*., 2022). Moreover, the improved rootstock root systems can better adapt to adverse environments, promote the proliferation of beneficial microorganisms, and enhance soil fertility and structure. Despite the labor‐intensive, time‐consuming, and contamination‐prone nature of traditional transgenic root production methods, recent research suggests that cutting plants and encouraging rooting can accelerate the growth of transgenic roots (Ma *et al*., 2022). However, the cutting approach requires strict management conditions and may lead to delays in the cultivation and dissemination of genetically improved rootstocks.

In summary, HPPB encompasses the following three main steps (Part I of Figure 1): First, the transgenic binary vector plasmid carrying the target gene is introduced into *A. rhizogenes* K599. Subsequently, K599 is cultured in YEP medium until the optical density at 600 nm (OD_600_ value) reaches a range of 0.6–0.8. Then, K599 is harvested and resuspended in MES solution (10 mM MgCl_2_, 10 mM MES [pH 5.6], and 100 μM AS), followed by incubation in the dark for 2–4 h to activate the root‐inducing function of *A. rhizogenes*. Second, select citrus plants aged 2–3 years. After removing thorns and branches from the stem, make precise incisions on the stem with a blade (Figure S7a), ensuring that each incision is deep enough to expose the phloem and reach the xylem layer. Subsequently, attach absorbent paper soaked with the MES solution containing K599 to the wound and keep it there for 20 min. Finally, cover the wound area with a HPPB box pre‐filled with 0–6 mm cultivation substrate (PINDSTRUP SPHAGNUM, Shanghai, China), and inject 1–2 mL of the MES solution containing K599 into the HPPB box. During the HPPB process, the grey matte HPPB box with a relatively wide range of applications is preferably selected (Figure S4).

**Figure 1:**
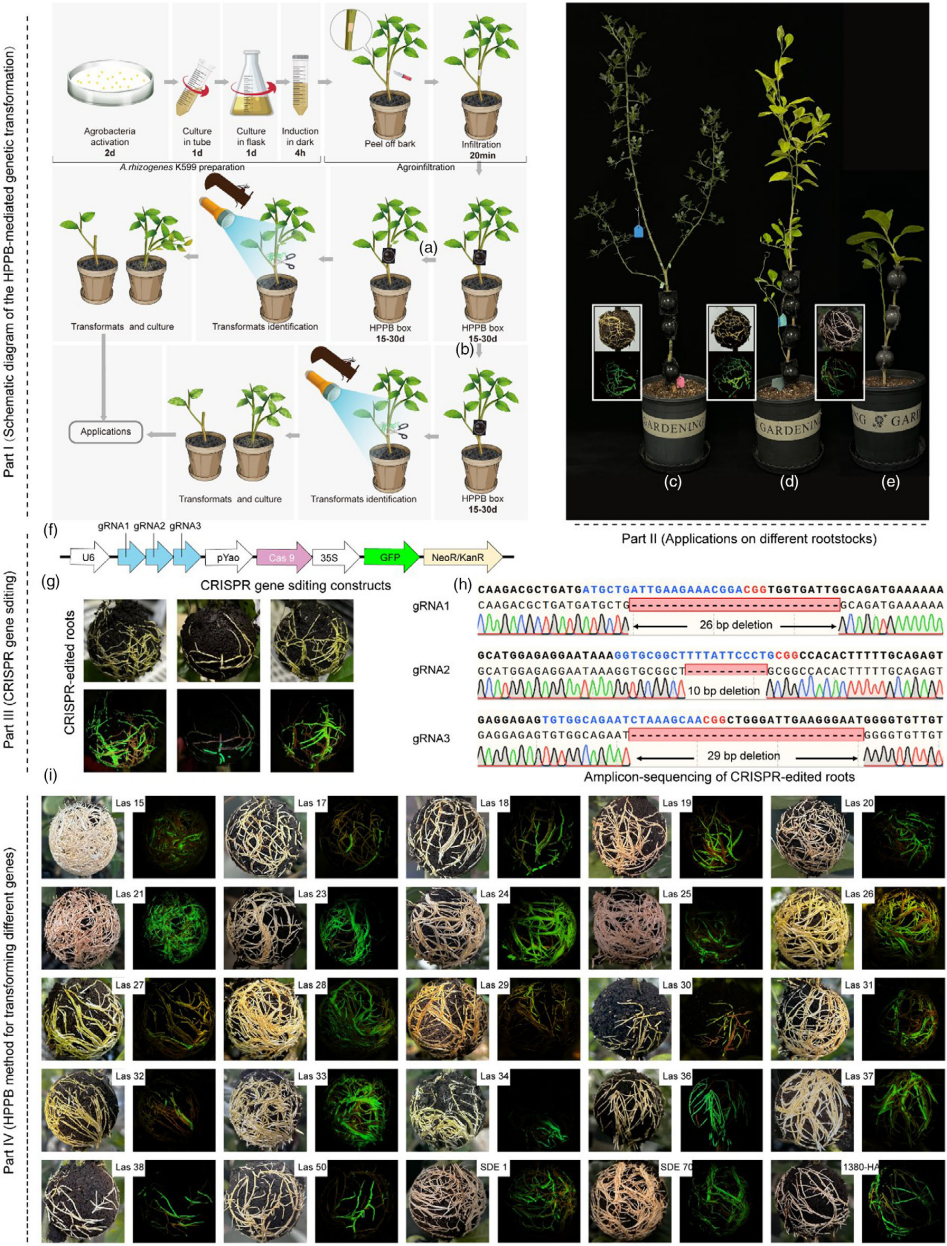
HPPB‐mediated genetic transformation: from operational workflow to practical applications. Part I Schematic diagram of HPPB‐mediated genetic transformation: (1) preparation of the plant for infection with the transgenic K599 inoculum; (2) HPPB genetic transformation; (3) (a) acquisition of materials with transgenic roots infected by pathogens; (b) acquisition of materials with transgenic roots. Detection and further cultivation of the genetically transformed roots. Part II HPPB in different rootstocks; (c) *Poncirus trifoliata* (L.) Raf.; (d) *Citrus* × *aurantifolia* (Christm.) Swingle; (e) *Citrus medica* L. Part III (f–h) Application of HPPB in CRISPR gene editing. Part IV (i) HPPB method for transforming different genes.

Then all plants were cultured in the greenhouse (16 h light/8 h dark, temperature maintained at 25–30 °C and humidity above 80%). The transgenic roots start to sprout within 2 weeks post‐genetic transformation, and a substantial number of transgenic roots can be obtained after 1–2 months of cultivation. The binary vector used for *A. rhizogenes* transformation carries a green fluorescent protein (GFP) tag (Figure S1a). This enables the preliminary screening of transgenic roots using a handheld ultraviolet fluorescent lamp under excitation/emission wavelengths 440/500 nm (Luyor‐3415RG, Shanghai, China). Additionally, a visible RUBY tag can be constructed (He *et al*., 2020), for the preliminary visual screening of red transgenic roots (Figure S1b). Subsequently, Western blotting (WB) and reverse transcription‐quantitative polymerase chain reaction (RT‐qPCR) can be employed to analyse the gene expression in transgenic roots (Figures S2a and S10). Typically, the validated genetically transformed roots can be excised and transplanted to establish new rootstock plants (Figure 1b). Within the HPPB process, we further integrated the grafting procedure to introduce systemic diseases (Figure 1a, Figure S7b), such as citrus huanglongbing (HLB) caused by *Candidatus* Liberibacter asiaticus (CLas). This approach enables concurrent acquisition of genetically transformed roots and disease infection (Figure S11), thereby saving time and costs for diseases with extended infection cycles, and offering a valuable research model for investigating gene functions associated with pathogenic mechanisms.

This study validated several common molecular biological applications in gene function studies. For example, by validating the differential expression patterns of promoters between CsSUC2 (Stadler and Sauer, 2019) and the 35S promoters fused with a GUS reporter gene, the paraffin sectioning and staining results revealed that CsSUC2 drove GUS expression in a phloem‐restricted region (Figure S1a). Additionally, the genome editing applied in HPPB was also confirmed by designing three concatenated gRNAs targeting the CDS region of gene *Cme102450* in a CRISPR‐Cas9 gene editing vector (Figure 1f–h) the salicylic acid hydroxylase SahA (NCBI: CP159585.1) of CLas was over‐expressed in the roots of *Citrus medica* L. via HPPB. Quantitative Analysis of Plant Hormones Results showed the salicylic acid content in transgenic roots was significantly decreased when compared with the control group (Figures S3 and S8). The application scope of the HPPB method is not limited to the above but holds significant implications for advancing molecular biology research and establishing economically efficient citrus rootstocks breeding programs.

## Conflict of interest

All authors declare no competing interests.

## Author contributions

Conceptualization: SD; writing – original draft preparation: SY, AY; writing – review and editing: SD; supervision: SD, ML; experiment and plant maintenance: SY, RF, YX, TT; data analysis: SY, AY; funding acquisition: SD, NW, ML. All authors contributed to the article and approved the submitted version.

## Supporting information


**Figure S1** Effects of different screening labels on large‐scale screening of rootstocks with genetic transformation in citrus (GFP/RUBY/GUS).
**Figure S2** *A. rhizogenes* K599‐mediated protein expression and subcellular localization.
**Figure S3** The salicylic acid content in roots is influenced by the *SahA* gene of the clas.
**Figure S4** Varieties of high‐pressure propagation boxes.
**Figure S5** High‐pressure propagation and verification of GFP‐transgenic root systems in other citrus rootstock varieties.
**Figure S6** The practical operation process and identification procedure illustration of HPPB technology for genetically transformed root systems.
**Figure S7** Optimizing HPPB transformation efficiency and exploring its applications.
**Figure S8** Verification of *SahA* transgenic roots.
**Figure S9** Applications of HPPB transgenic roots in subcellular localization.
**Figure S10** Gene expression levels in the HPPB – transgenic roots.
**Figure S11** HPPB transgenic roots obtained from plants infected with Huanglongbing (HLB) are also susceptible to the disease.
**Figure S12** HPPB is applicable to different citrus species.
**Table S1** The genetic transformation effects of HPPB on different citrus variety rootstocks.
**Table S2** Primer sequence.
**Table S3** Comparison table of transformation rates using different *A. rhizogenes* strains with HPPB.
**Table S4** Comparison of rootstock genetic transformation methods (*Citrus sinensis* Osb. × *Poncirus trifoliata* (L.) Raf.).
**Table S5** HPPB application in genetic transformation efficiency statistics for different genes.

## Data Availability

The data that supports the findings of this study are available in the supplementary material of this article.
