# Supplementary material for: Establishment and application of high‐pressure propagation breeding (HPPB)‐mediated genetic transformation system in citrus rootstocks
Source: Plant Biotechnol J. 2025 Apr 29;23(7):2790–2. doi: 10.1111/pbi.70072 (PMC12205874; doi:10.1111/pbi.70072)
Supplement: Supplementary file 1 — Figure S1 Effects of different screening labels on large‐scale screening of rootstocks with genetic transformation in citrus (GFP/RUBY/GUS). Figure S2 A. rhizogenes K599‐mediated protein expression and subcellular localization. Figure S3 The salicylic acid content in roots is influenced by the SahA gene of the clas. Figure S4 Varieties of high‐pressure propagation boxes. Figure S5 High‐pressure propagation and verification of GFP‐transgenic root systems in other citrus rootstock varieties. Figure S6 The practical operation process and identification procedure illustration of HPPB technology for genetically transformed root systems. Figure S7 Optimizing HPPB transformation efficiency and exploring its applications. Figure S8 Verification of SahA transgenic roots. Figure S9 Applications of HPPB transgenic roots in subcellular localization. Figure S10 Gene expression levels in the HPPB – transgenic roots. Figure S11 HPPB transgenic roots obtained from plants infected with Huanglongbing (HLB) are also susceptible to the disease. Figure S12 HPPB is applicable to different citrus species. Table S1 The genetic transformation effects of HPPB on different citrus variety rootstocks. Table S2 Primer sequence. Table S3 Comparison table of transformation rates using different A. rhizogenes strains with HPPB. Table S4 Comparison of rootstock genetic transformation methods (Citrus sinensis Osb. × Poncirus trifoliata (L.) Raf.). Table S5 HPPB application in genetic transformation efficiency statistics for different genes. [file PBI-23-2790-s001.pdf]

Supplementary Data:

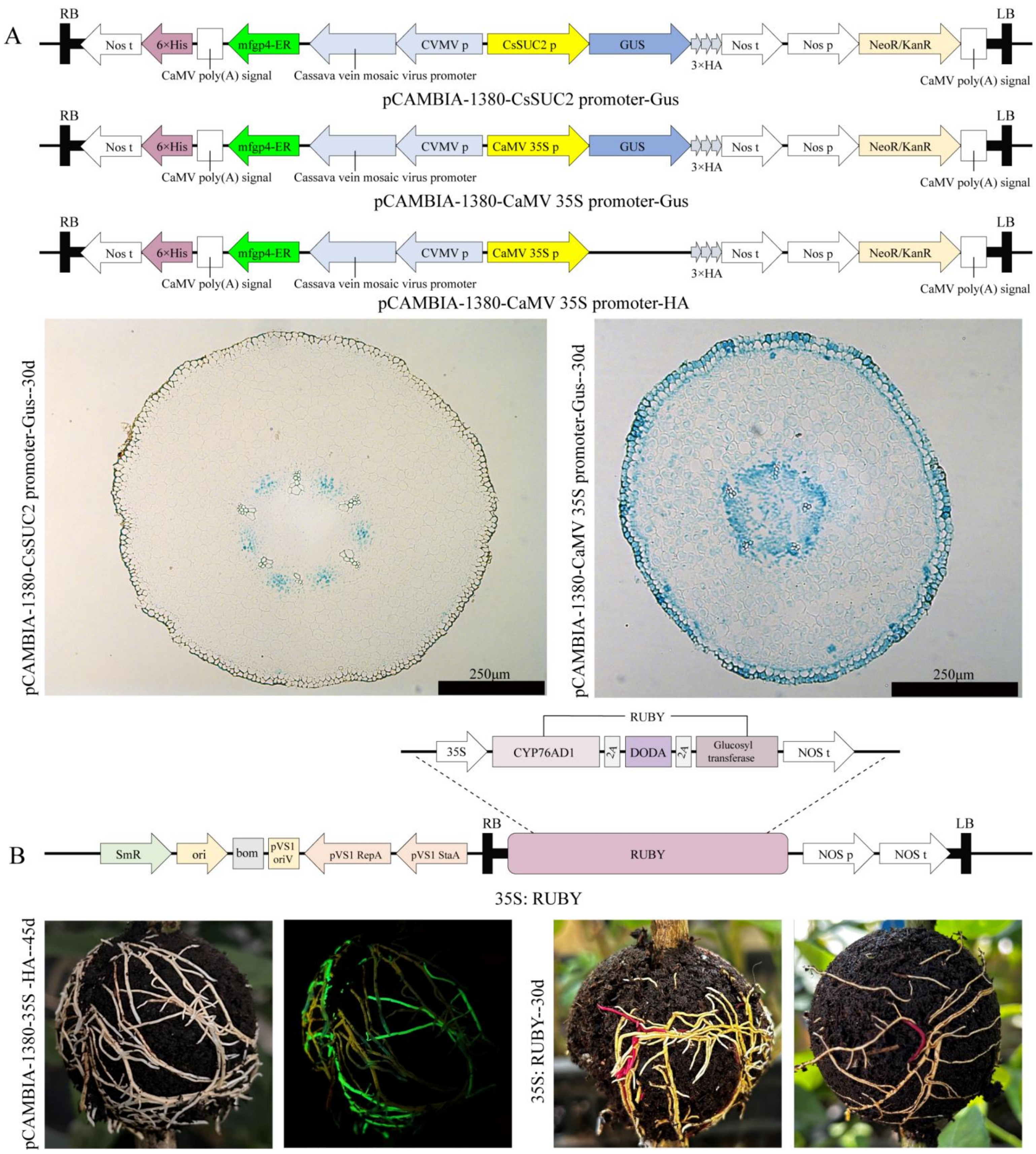

Figure S1. Effects of Different Screening Labels on Large-Scale Screening of Rootstocks with Genetic Transformation in Citrus(GFP/RUBY/GUS)

A) The diagram shows the profiles of three plasmids fragments constructed based on the pCambia-1380 vector backbone, from top to bottom: the citrus phloem-specific CsSUC2 promoter plasmid carrying the Gus reporter gene tag, the 35s strong promoter plasmid with the Gus reporter gene tag, and the empty vector of the 35s strong promoter pCambia-1380. The genetically transformed root systems obtained from the first two plasmids were well observed for gene-specific expression in different root tissues using the paraffin section Gus staining method, and the application effect of the Gus tag is shown in the lower figure. The 35s strong promoter pCambia-1380 empty vector high-pressure propagation ball photographed at 45 days is in the lower left of this figure, showing the application effect of the GFP tag.

B) The diagram shows the Ruby vector map, and the lower high-pressure propagation ball shows the application effect of the Ruby tag at 30 days.

Supplementary Data:

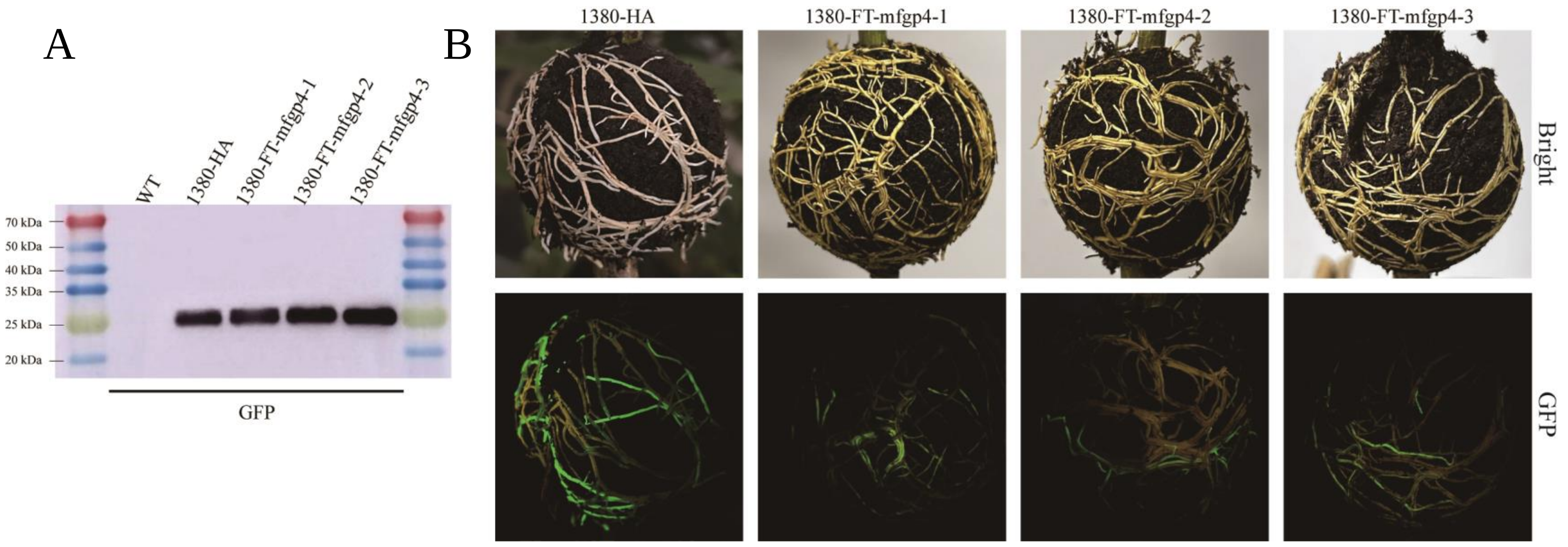

Figure S2. *A. rhizogenes* K599-Mediated Protein Expression and Subcellular Localization

A) Western blot diagram with GFP antibody;

B) Corresponding transgenic high-pressure propagation spheres.

A

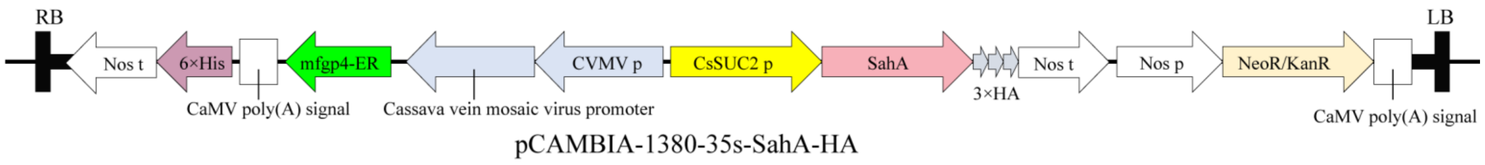

B

	Sample information			Test Concentration(ng/mL)			Sample Content(ng/g)		
Name	Number	Weight(g)	Volume(mL)	SA	MESA	GA1	SA	MESA	GA1
1380-HA-1	D1	0.1249	0.2	57.850	n.a	1.690	92.634	n.a	2.706
1380-HA-2	D2	0.1164	0.2	57.010	n.a	1.670	97.955	n.a	2.869
1380-HA-3	D3	0.1185	0.2	57.160	n.a		96.473	n.a	2.498
1380-HA-4	D4	0.1234	0.2	57.080	n.a	1.630	92.512	n.a	2.642
SahA-1	D5	0.2055	0.2	1.180	n.a	0.040	1.148	n.a	0.039
SahA-2	D6	0.2016	0.2	1.080	n.a	0.040	1.071	n.a	0.040
SahA-3	D7	0.2476	0.2	1.530	n.a	0.030	1.236	n.a	0.024
SahA-4	D8	0.2554	0.2	1.590	n.a	0.050	1.245	n.a	0.039
Blank	K	0	0.2	0		0			

Figure S3. The Salicylic Acid Content in Roots is Influenced by the *SahA* Gene of the Clas

A) Schematic diagram of the pCamnia 1380-35s-SahA-HA vector construction.

B) Hormone level measurements in roots with the pCambia-1380 empty vector and *SahA* genetically modified roots.

Supplementary Data:

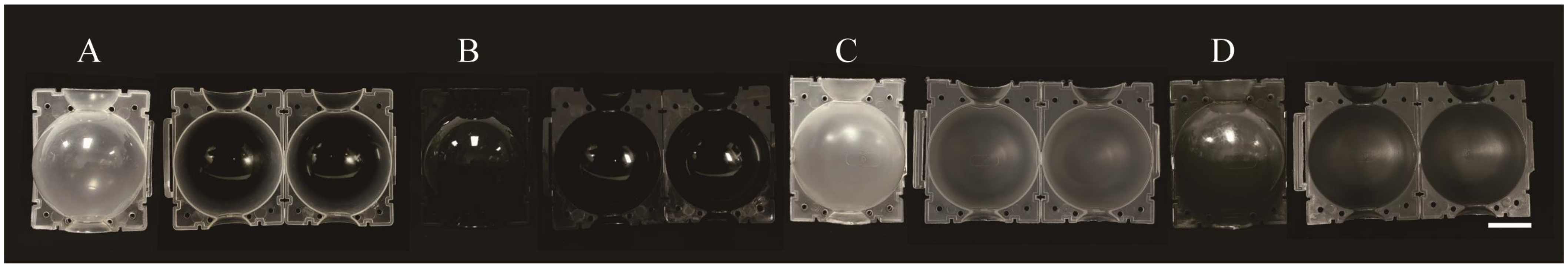

Figure S4: Varieties of High-Pressure Propagation Boxes.
This figure displays the open and closed states of four distinct HPPB boxes, each exhibiting unique color and texture variations. A) Transparent Glossy - A clear, shiny HPPB box. B) Black Glossy - A dark, smooth HPPB box. C) Transparent Matte - A clear, matte-finished HPPB box. D) Gray Matte - A subtly colored, matte-finished HPPB box. A scale bar indicating 2 cm is provided for reference. The boxed end with teeth firmly attaches to the stem's base, while the top can be sealed using a membrane to reduce water evaporation.

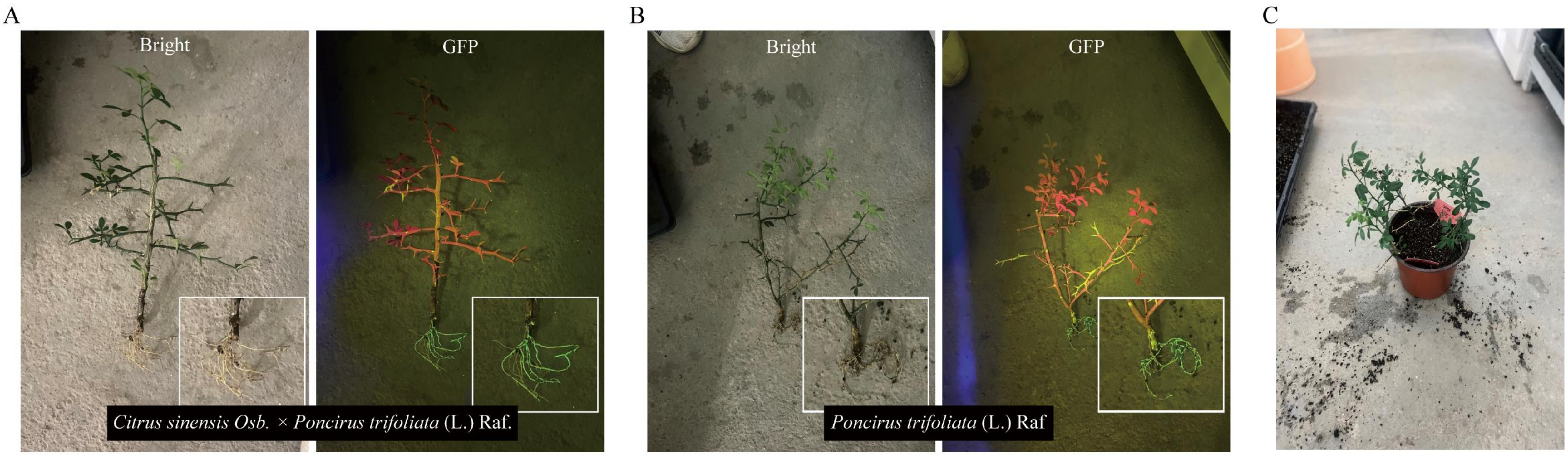

Figure S5: High-Pressure Propagation and Verification of GFP-Transgenic Root Systems in Other Citrus Rootstock Varieties
Following the acquisition of genetically modified root systems through high-pressure propagation, the *Citrus sinensis* Osb. × *Poncirus trifoliata* (L.) Raf. (A) and *Poncirus trifoliata* (L.) Raf (B) are cut below the propagation chamber. The upper stem sections are scrutinized under bright and with the LUYOR-34145RG Hand-Held lamp to confirm the integration of GFP-transgenic roots. (C) The stem portions that exhibit GFP-transgenic roots are transferred to pots for advanced root studies. The remaining lower stem sections are preserved for the subsequent round of high-pressure propagation.

Supplementary Data:

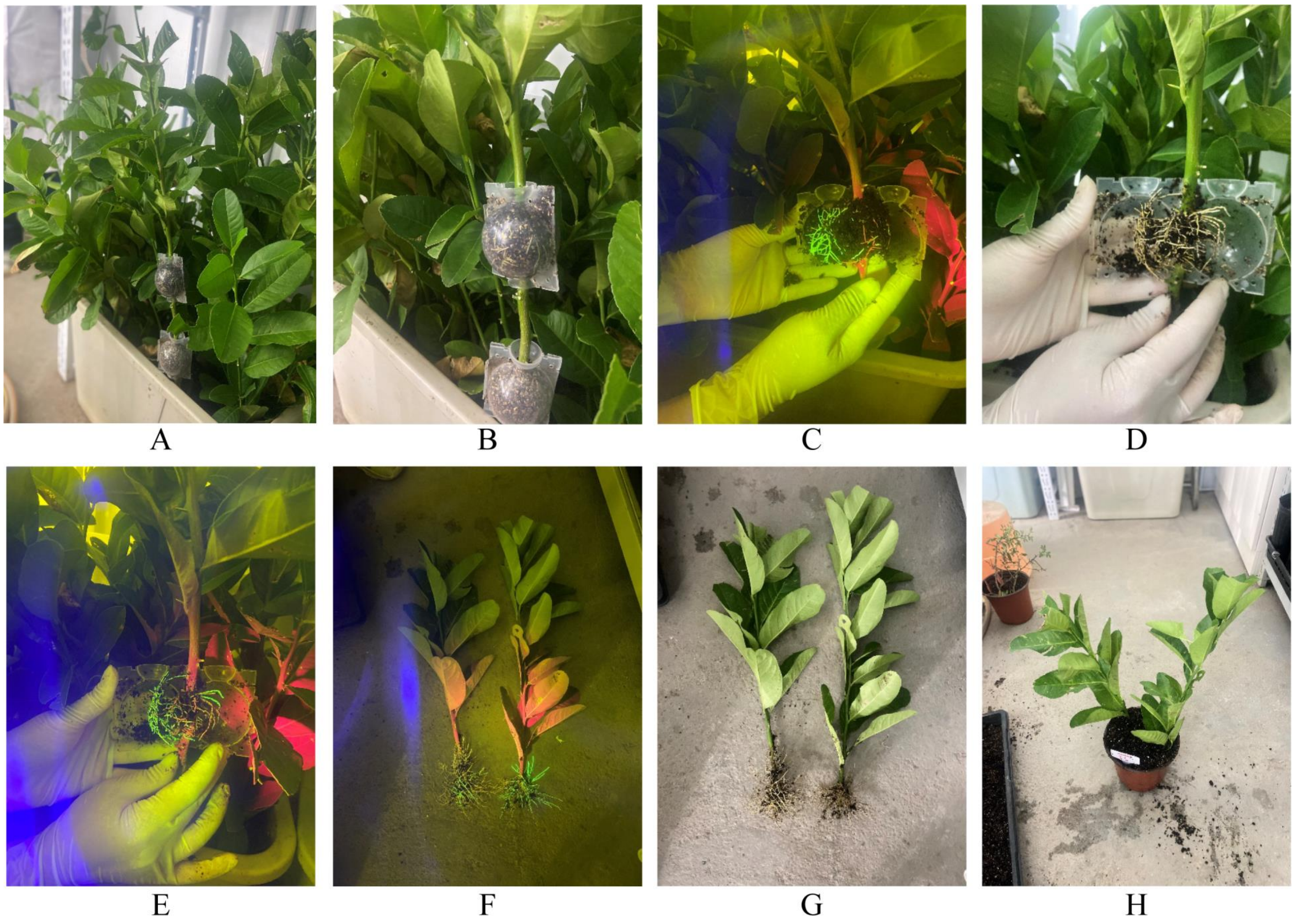

Figure S6: The Practical Operation Process and Identification Procedure Illustration of HPPB Technology for Genetically Transformed Root Systems.

HPPB workflow for 3-year-old *Citrus medica* L.. A) Select 3-year-old seedlings for HPPB experiments. B) Utilize transparent HPPB boxes (Supplementary Figure 1) to facilitate monitoring of root development. C) Perform a preliminary screening of genetically transformed roots using a the LUYOR-34145RG Hand-Held lamp. D) After soil removal, assess root development to determine suitability for transplantation. E) Further verify the transformation rate and efficacy through fluorescence detection. F) Collect transformed roots for sequencing validation and process non-transformed roots according to experimental objectives (green: transformed roots, white: non-transformed roots). G) Transplant when transformed roots exceed five in number and are at least 8 cm in length. H) Transplant into well-draining and breathable soil to continue cultivation, directly obtaining genetically transformed rootstock plants of over three years old.

Supplementary Data:

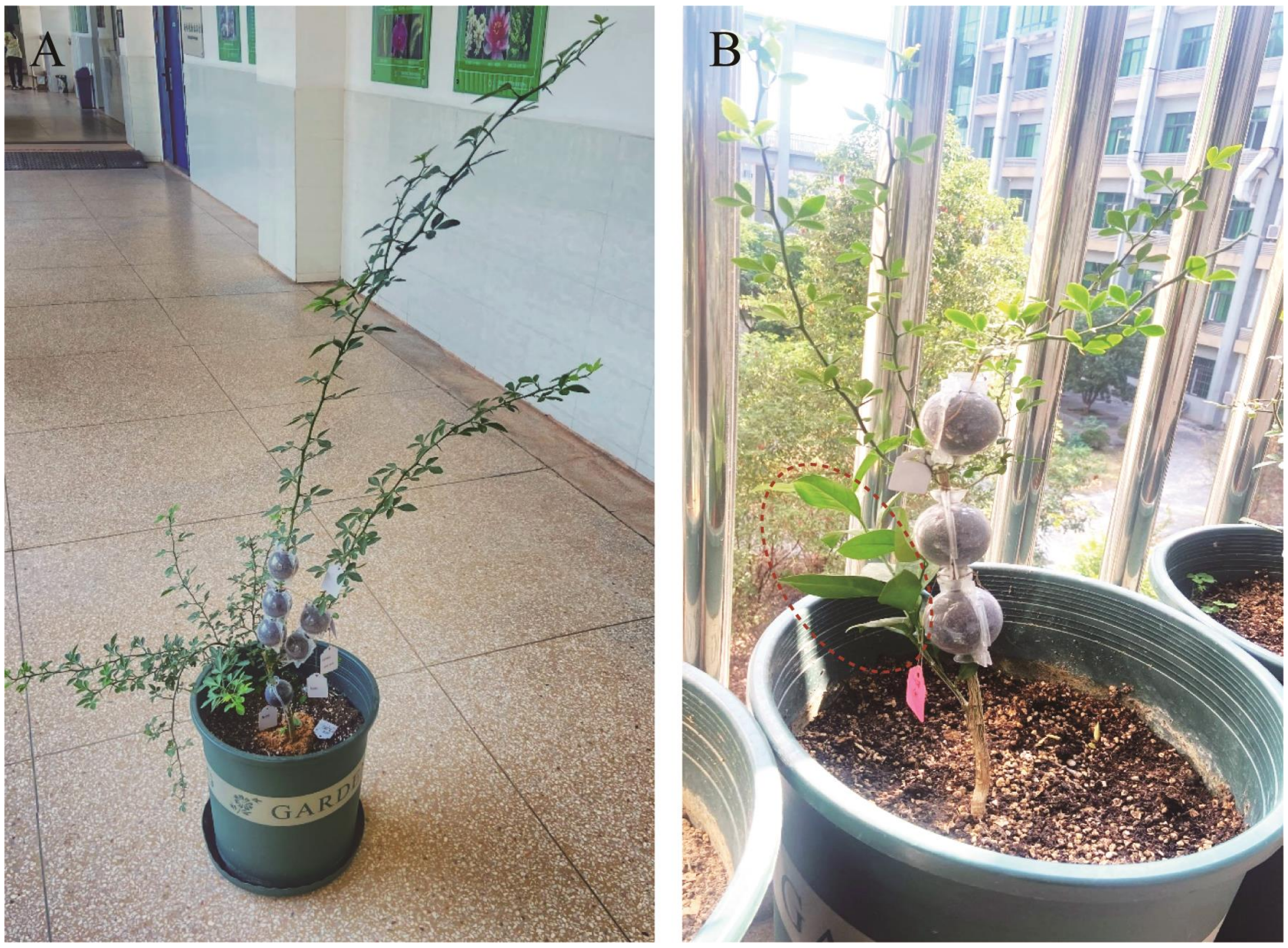

Figure S7: Optimizing HPPB Transformation Efficiency and Exploring Its Applications.
A) Application of HPPB on 3-year-old *Poncirus trifoliata* (L.) Raf. Depending on the stem conditions of different citrus plants, efficient transformation of 2 to 6 HPPB boxes can be achieved. B) 3-year-old *Poncirus trifoliata* (L.) Raf demonstrate the combined application of grafting and HPPB. The area enclosed by the red dashed line indicates the part of the grafting transmission, which uses *Citrus sinensis* (L.) Osbeck infected with Citrus Huanglongbing disease.

Supplementary Data:

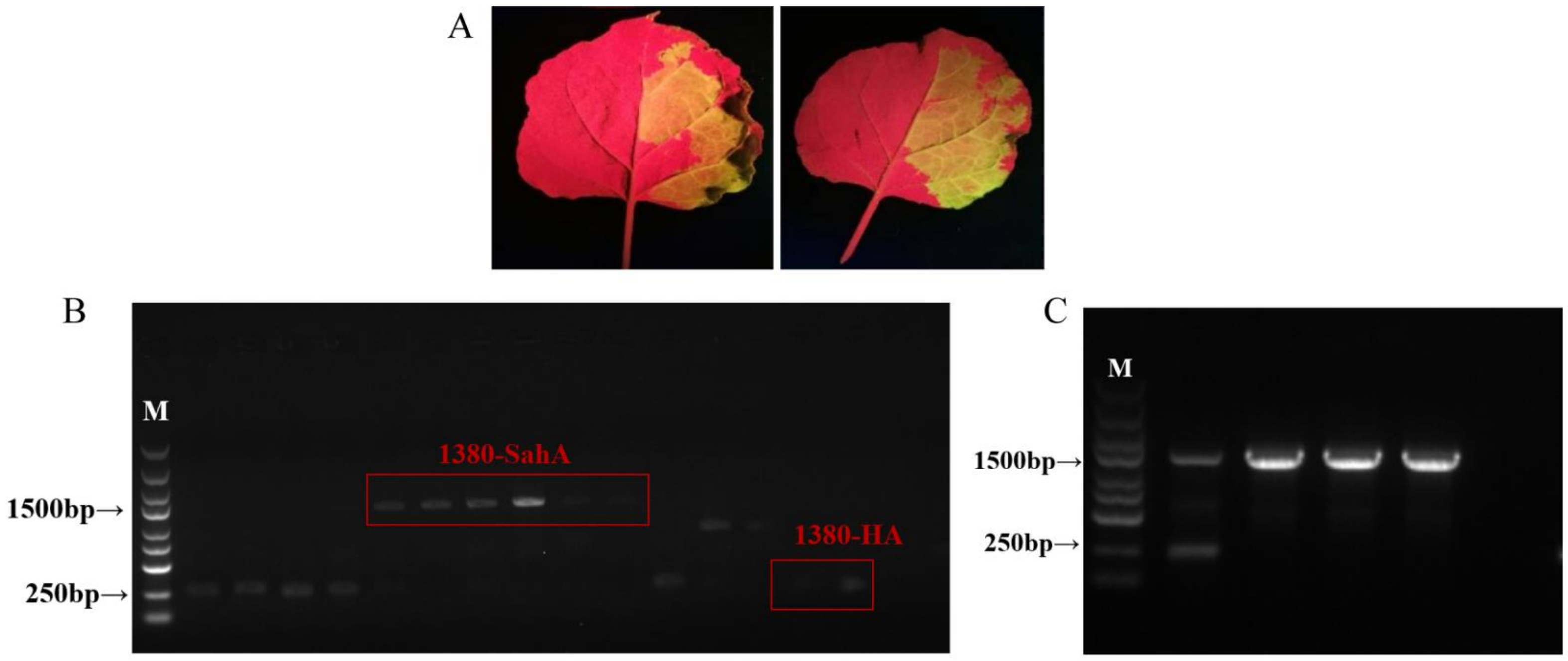

Figure S8: Verification of *SahA* Transgenic Roots.

A) Formulation and application of infection solution for tobacco transformation verification post-transformation of K599 with pCambia1380-SahA-HA and pCambia1380-HA vector. B) DNA extracted from the transgenic roots was used to amplify the target gene *SahA* (1293bp) with vector primers F/R-1380HA-EV, resulting in the expected band size, with the control empty vector producing a 181bp band. For comprehensive gene sequencing, the PCR product was cloned into the pEASY-Blunt vector (Trans, CB01) and transformed into DH5α for plasmid template extraction. C) Agarose gel electrophoresis using pEASY-Blunt vector's universal M13F/R primers revealed bands matching the *SahA* gene size, and the plasmid was purified for sequencing.

Supplementary Data:

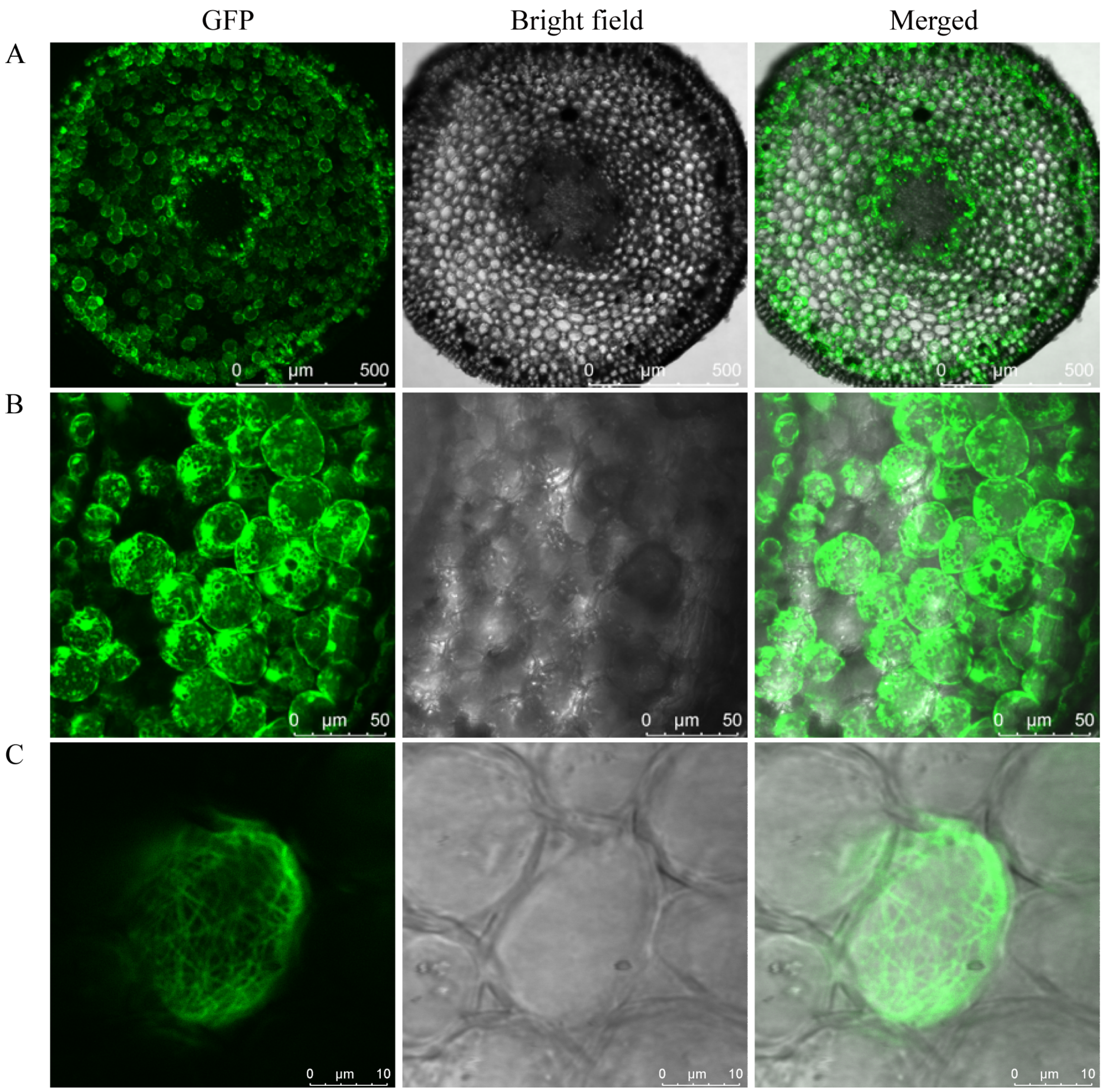

Figure S9: Applications of HPPB Transgenic Roots in Subcellular Localization.

This figure demonstrates the application of HPPB genetically transformed roots in subcellular localization studies, specifically regarding the localization of the endoplasmic reticulum (A, B) and microfilaments (C). The protein used for endoplasmic reticulum localization is GFP - ER (mfgp4-ER). Its information is as follows: Synthetic construct modified green fluorescent protein GFP-ER (mfgp4-ER) mRNA, complete cds Sequence ID: U87625.1 Length: 818 Number of Matches: 1. The construction vector is pCambia-1380. The scale bar is in the bottom-right corner of each figure.

A) A fresh cross-section image of HPPB genetically transformed roots taken by a fluorescence microscope, which shows the distribution of the GFP-ER (mfgp4-ER) protein.
B) The magnified image, which more clearly shows the subcellular localization of the GFP-ER (mfgp4-ER) protein.
C) GFP-Lifeact: This shows the subcellular localization of microfilaments. The vector used is pEarleyGate201-UBQ10pro::EGFP - ABD2, which was provided by Prof. Ming Chang from Nanjing Agricultural University. Genetically transformed roots were obtained through the HPPB method, and their fresh cross - sections were observed under a confocal microscope to obtain this subcellular localization image.

Supplementary Data:

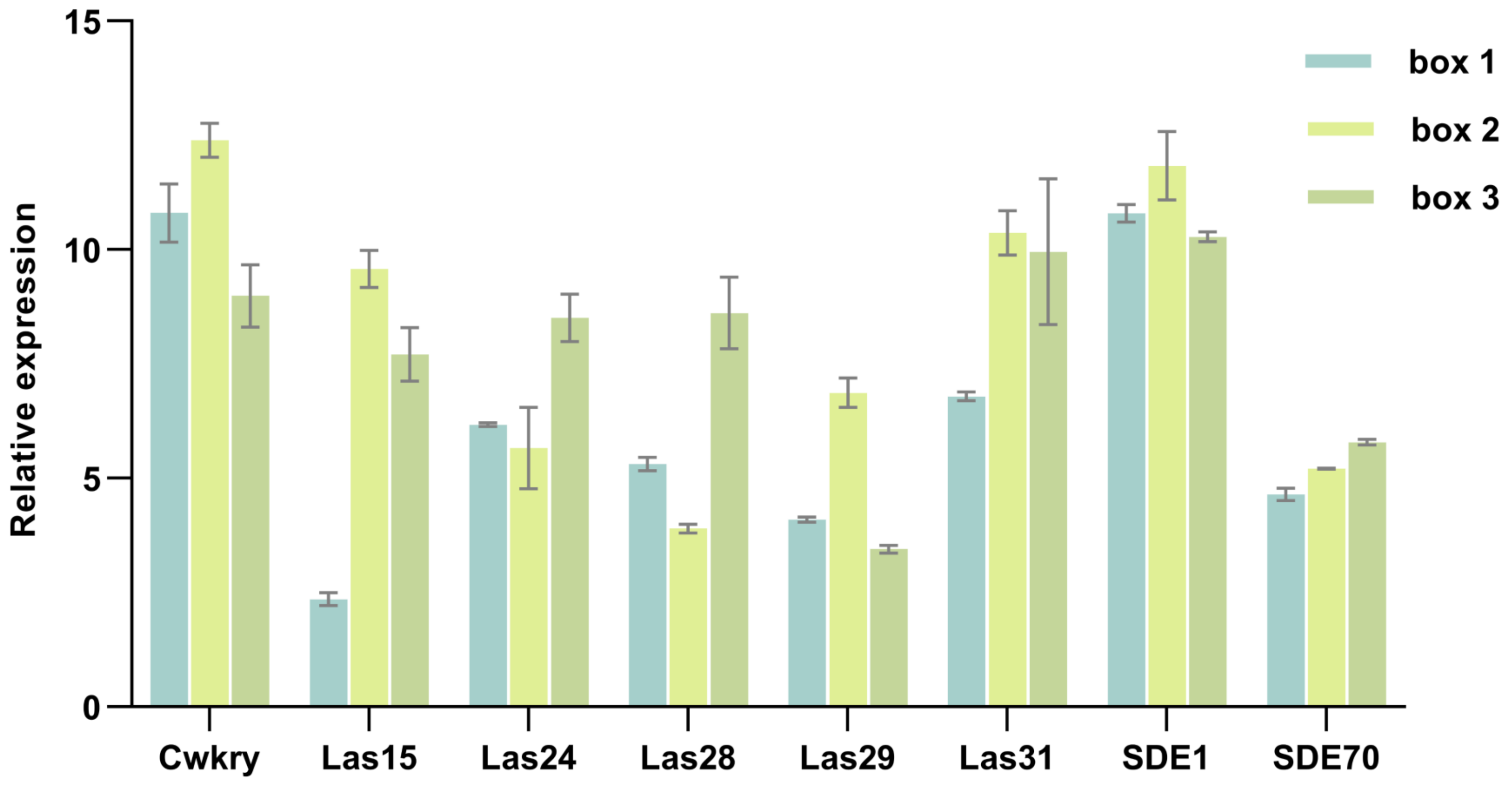

Figure S10: Gene Expression Levels in the HPPB - Transgenic Roots.
We obtained HPPB transgenic roots from *Citrus medica* L.. Subsequently, we extracted RNA from these roots and performed reverse transcription to successfully obtain cDNA. The primers used for detection are detailed in the attached Table S2(SEQ ID NO.9~ NO.24). It should be noted that the transgenic roots corresponding to box1, box2, and box3 were taken from different plants and different HPPB boxes respectively. All the genes to be detected were constructed on the pCambia1380 vector. As shown in the figure, genes from *Cwkry* to *SDE70* all exhibited relatively high expression levels in the HPPB transgenic roots.

Supplementary Data:

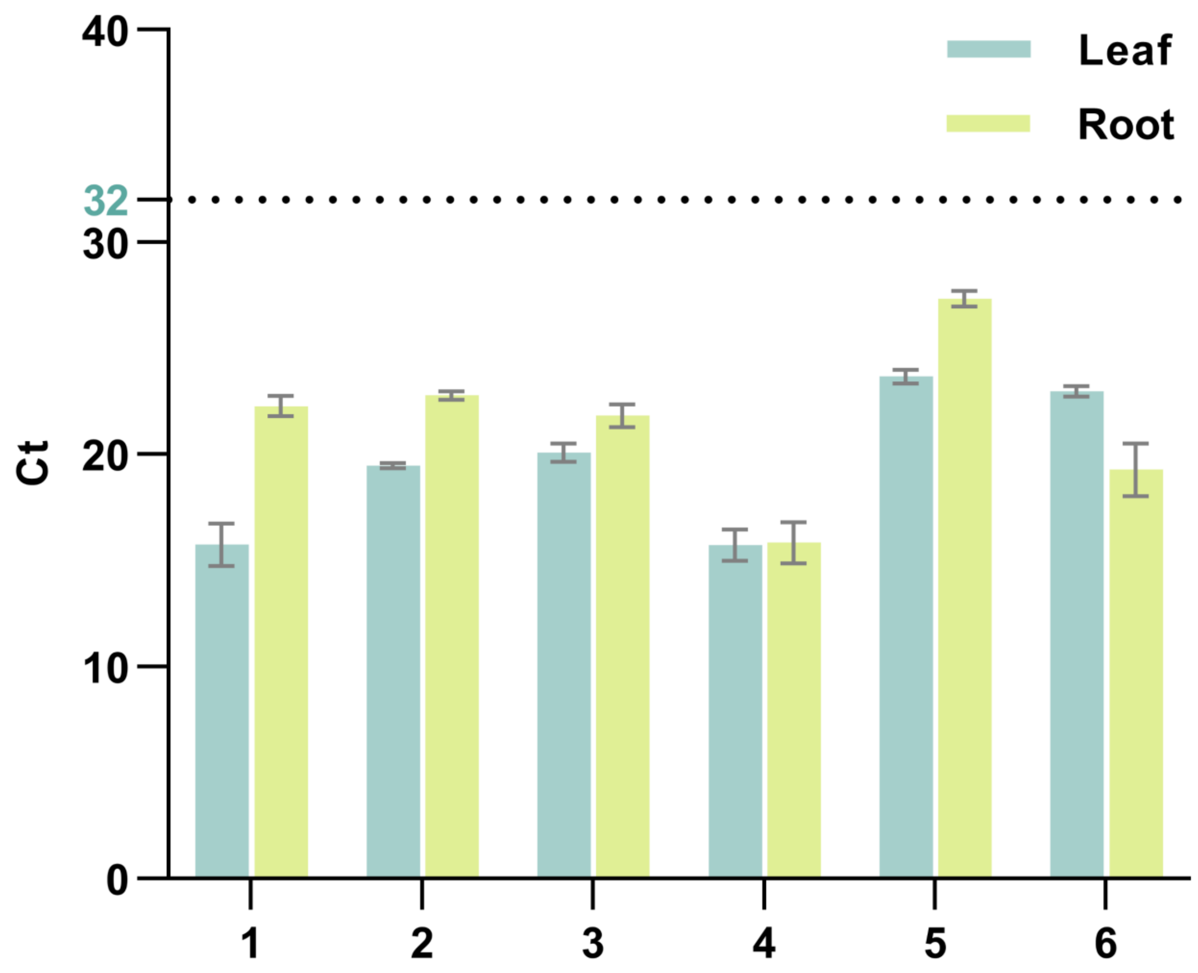

Figure S11: HPPB Transgenic Roots Obtained from Plants Infected with Huanglongbing (HLB) Are Also Susceptible to the Disease

In the detection of Huanglongbing (HLB) in citrus, the Ct value is a crucial detection indicator. Generally, we extract the DNA from plant leaves for testing. When the detected Ct value is less than or equal to 32, it can be determined that the sample is infected with HLB. As shown in the figure, we selected 6 rangpur *Citrus limon (L.)* Burm. f plants infected with HLB, and extracted the DNA from their leaves and the corresponding HPPB roots respectively. Then we used the RNR primers (Table S2 SEQ ID NO.25~26) to measure the Ct values of the samples. The test results clearly indicate that the HPPB roots have been successfully infected with HLB.

Supplementary Data:

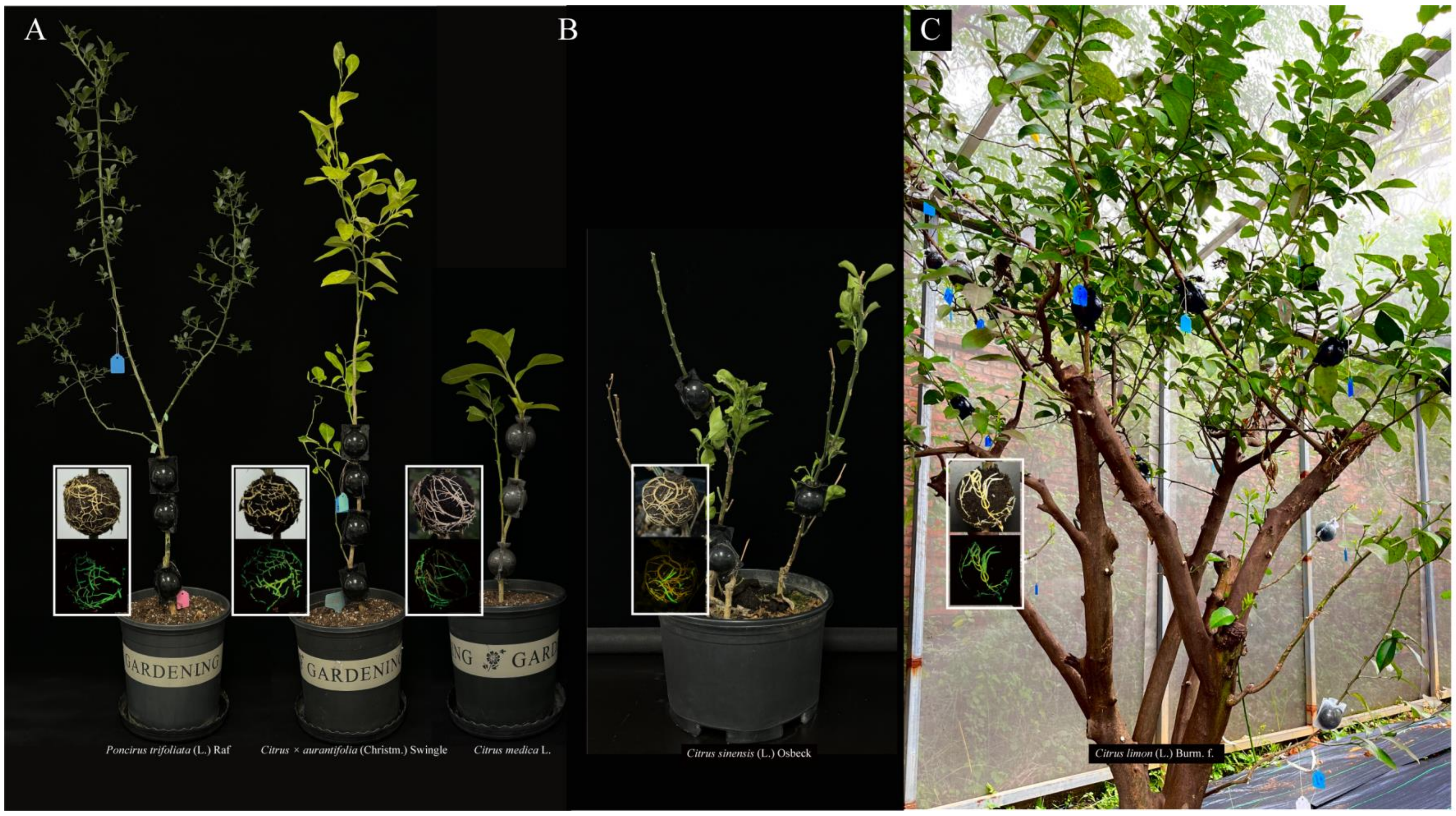

Figure S12: HPPB is Applicable to Different Citrus Species

A) HPPB applicable to different rootstocks.(*Poncirus trifoliata* (L.) Raf, *Citrus ✕ aurantifolia* (Christm.) Swingle, *Citrus medica* L.)

B) HPPB applicable to *Citrus sinensis* (L.) Osbeck difficult to transform.

C) HPPB applicable to field applications. (*Citrus limon* (L.) Burm. f.)

Supplementary Data:

Table S1: The genetic transformation effects of HPPB on different citrus variety rootstocks.

Stock variety	Rooting rate%	Transformation efficiency%	Number of balls	Number of root-generating balls	Number of transformed balls
Citrus medica L.	95%	82.22%	95	90	74
Citrus limon (L.) Burm. f.	93%	80.35%	120	112	90
Citrus sinensis Osb. × *Poncirus trifoliata* (L.) Raf.	72%	54.33%	176	127	69
Poncirus trifoliata (L.) Raf	69%	50.26%	274	189	95

Rooting rate%=Number of root-generating balls/Number *100%
Transformation efficiency%=Number of successfully transformed balls/Number of root-generating balls*100%

Table S2: Primer sequence

Name	Primer sequence（5'→3'）	Number*
SahA-F	AGAACACGGGGGACATGATAATCGGAGCAG	SEQ ID NO.1
SahA-R	caggaacgtcgtatgggtaTTCGGGTATTTTATATTGATAG	SEQ ID NO.2
F-1380HA-EV	CAATCCCACTATCCTTCGCA	SEQ ID NO.3
R-1380HA-EV	catcatacggatatagggat	SEQ ID NO.4
M13F	GTAAAACGACGGCCAGT	SEQ ID NO.5
M13R	CAGGAAACAGCTATGAC	SEQ ID NO.6
CsPDT Cas9-F	ATGAACGGCATAGAGAGGAGG	SEQ ID NO.7
CsPDT Cas9-R	CGAACGGTTGCAAATAGGACAAG	SEQ ID NO.8
Cwkry_F	GAGCACAACCATCCACTTCCTT	SEQ ID NO.9
Cwkry_R	AAGAAGCCATCTGTTCCACCAA	SEQ ID NO.10
Las 15_F	AGTCGGAACCTTGACGTATAGC	SEQ ID NO.11
Las 15_R	ACCTGCCCATCTCCAAATTGAT	SEQ ID NO.12
Las 24_F	TCTACGGCGATGACGGAGGA	SEQ ID NO.13
Las 24_R	GACGATCTGCTTCTGGCAACTG	SEQ ID NO.14
Las 28_F	GCACCGAGATTGTATGGCTTGA	SEQ ID NO.15
Las 28_R	CAATAGATCACGAGCGGACACT	SEQ ID NO.16
Las 29_F	TAGATCGGATACCAGCAAGAGG	SEQ ID NO.17
Las 29_R	ATACGCACACGACGGACAC	SEQ ID NO.18
Las 31_F	CAATGATGGTGGCTATGGATGT	SEQ ID NO.19
Las 31_R	CATATGAGTTGCACGCAGTTGT	SEQ ID NO.20
SDE 1_F	GCGTGTTCCTGATGTCTCTGAA	SEQ ID NO.21
SDE 1_R	CGAGTGCGACTAATGTGCTTCT	SEQ ID NO.22
SDE 70_F	GGCATGGGAAATATACCGAGGT	SEQ ID NO.23
SDE 70_R	CGTTCGATGGCATGTCCTTGTA	SEQ ID NO.24
RNR_F	CATGCTCCATGAAGCTACCC	SEQ ID NO.25
RNR_R	GGAGCATTTAACCCCACGAA	SEQ ID NO.26

* SEQ ID NO.1~6 Figure S8 primers, SEQ ID NO.7~8 Figure1 Part III Crisper sequencing primers, SEQ ID NO.9~24 Gene expression level RT-qPCR primers, SEQ ID NO.25~26 HLB qPCR primers for disease detection.

Supplementary Data:

Table S3:Comparison Table of Transformation Rates Using Different *A. rhizogenes* Strains with HPPB

Group	*A. rhizogenes* Strains	Genotype	Number of root-generating balls	Number of transformed balls	Transformation efficiency%
Contrast experiment 1	ATCC15834	*A. rhizogenes* pRi15834 (agropine type)	90	51	56.67%
Contrast experiment 2	C58C1	*A. rhizogenes* pRiA4b (agropine type)	100	44	44%
Experiment 1	K599	*A. rhizogenes* pRi2659 (agropine type)	80	66	82.5%
Contrast experiment 3	Ar.Qual	*A. rhizogenes* Ar Qual Ri (agropine type)	80	46	57.5%

Transformation efficiency%=Positive number/Number*100%. The genotype of all strains agropine type

Table S4:Comparison of Rootstock Genetic Transformation Methods (*Citrus sinensis Osb.* ╳ *Poncirus trifoliata* (L.) Raf.)

Methods	Cultivation cycle for seedlings (3-year-old saplings)	Transformation efficiency%	Problem
A.tumefaciens-mediated stem segment infection for genetic transformation.	4 years (1 year for genetic transformation, 3 years for the juvenile period).	2‰-5‰	contamination and Time consuming
A.rhizogenes-mediated cutting induction for genetic transformation.	3-4 years (2 months for genetic transformation, 2-3 years for the juvenile period).	37%	Time-consuming
HPPB-mediated genetic transformation.	5 months (2 months for genetic transformation, 3 months for the juvenile period).	82%	

Table S5: HPPB Application in Genetic Transformation Efficiency Statistics for Different Genes

SDE number	Protein ID	Total number of balls	Number of root-generating balls	Number of successfully transformed balls
Las 15	WP_015824940.1	6	6	6
Las 17	WP_015824938.1	6	6	6
Las 18	WP_012778429.1	8	7	7
Las 19	WP_015452986.1	7	7	7
Las 20	WP_015453017.1	6	5	5
Las 21	WP_244392125.1	7	7	7
Las 23	WP_012778434.1	7	7	7
Las 24	WP_040055304.1	8	8	8
Las 25	WP_015824948.1	6	6	6
Las 26	WP_015452879.1	6	5	3
Las 27	WP_012778603.1	6	4	4
Las 28	WP_012778634.1	6	4	4
Las 29	WP_012778645.1	6	5	5
Las 30	WP_015452616.1	8	8	8
Las 31	WP_015452630.1	8	8	8
Las 32	WP_015452419.1	7	7	7
Las 33	WP_012778374.1	7	5	5
Las 34	WP_015452743.1	7	7	7
Las 36	WP_015452812.1	7	6	6
Las 37	WP_015452988.1	7	6	6
Las 38	WP_015452848.1	6	4	4
SDE 1	WP_015452985.1	5	5	5
SDE 15	WP_015452737.1	7	7	7
SDE 70	WP_015452552.1	7	7	7
p1380-HA	——	6	6	6
Total		167	153	151
